# Silk Fibroin Sheets Improve the Strength of Colon Anastomoses in Wistar Rats

**DOI:** 10.3390/jfb17030126

**Published:** 2026-03-04

**Authors:** Mohamed Hassin Mohamed Chairi, Francisco José Huertas Peña, Jorge García-García, Laura López-Escánez, Salvador D. Aznar-Cervantes, Patricia Becerra Massare, Julio Gálvez, Per Anderson, José Alberto Molina-Tijeras, María Elena Rodríguez-Cabezas

**Affiliations:** 1 Servicio de Cirugía General, Hospital Universitario Virgen de las Nieves, 18012 Granada, Spain; 2 Instituto de Investigación Biosanitaria de Granada (ibs.GRANADA), 18012 Granada, Spainjgalvez@ugr.es (J.G.); merodri@ugr.es (M.E.R.-C.); 3 Departmento de Farmacologia, Center for Biomedical Research (CIBM), University of Granada, 18071 Granada, Spain; 4 Departamento de Biotecnología, Genómica y Mejora Vegetal, Instituto Murciano de Investigación y Desarrollo Agrario y Medioambiental (IMIDA), La Alberca, 30150 Murcia, Spain; salvadord.aznar@carm.es; 5 Servicio de Anatomía Patológica, Hospital Universitario Clínico San Cecilio, 18014 Granada, Spain; 6 CIBER de Enfermedades Hepáticas y Digestivas (CIBER-EHD), Instituto de Salud Carlos III, 28029 Madrid, Spain; 7 Departamento de Bioquímica, Biología Molecular III e Inmunología, University of Granada, 18016 Granada, Spain; 8 Servicio de Análisis Clínicos e Inmunología, Hospital Universitario Virgen de las Nieves, 18012 Granada, Spain

**Keywords:** anastomotic leakage, colon, silkworm, fibroin sheets, wound healing, burst strength

## Abstract

Colorectal resection and subsequent anastomosis are the standard curative procedures for a variety of colorectal pathologies. However, anastomotic leakage (AL) is an early and frequent complication that can have life-threatening outcomes. The study aimed to evaluate the effect of silkworm fibroin sheets on colon anastomotic strength and wound healing early after intervention in Wistar rats. Male Wistar rats were randomized into two groups, control (N = 11) and fibroin (N = 11), and subjected to end-to-end colo-colic anastomosis. In the fibroin group, a single layer of fibroin membrane was applied externally around the anastomosis. The animals were sacrificed three days after the operation (POD3) and intestinal adhesions, anastomotic bursting pressure and histological parameters based on the eosin, hematoxylin, and Masson’s trichrome stains were compared between the groups. Fibroin-treated rats showed a significant increase in anastomotic bursting pressure compared to control animals (69 (18) vs. 41 (28) mmHg), whereas no differences in the intestinal adhesion scores were detected. No significant differences in the numbers of granulocytes, monocytes/macrophages and fibroblasts, nor the amount of collagen fibers, as measured by Masson’s trichrome stain, were found between the groups. These results indicate that fibroin sheets could represent a simple and promising tool to provide mechanical support and improve colonic anastomotic strength early after intervention.

## 1. Introduction

The classic surgical principles for a successful colo-colic and colorectal anastomosis are a well-nourished patient with no systemic disease, no fecal or purulent contamination, adequate exposure and access, gentle tissue management, absence of distal tension and obstruction, approximation of well-vascularized bowel ends, and meticulous surgical techniques. However, even if all the above is achieved, anastomotic leakage (AL) may still occur. The prevalence of AL is 2–20%, depending on the site of the anastomosis and a plethora of modifiable and nonmodifiable preoperative, intraoperative and perioperative risk factors [1,2]. AL rates have remained almost unchanged over the past decades, and most leaks occur during the first 5–10 days following surgery [3,4,5,6]. AL is associated with serious adverse outcomes, including a need for bowel stomas, nosocomial and organ or space infections, sepsis, and mortality [7]. AL after cancer surgery has also been associated with an increased risk of disease recurrence [8].

AL detected after postoperative day (POD) six is believed to be the result of failed wound healing rather than inappropriate surgical procedures [9,10]. The wound healing process of an anastomosis is divided into an early inflammatory phase (day 1–4) characterized by hemostasis and provisional closure meditated by platelets, neutrophils, and macrophages, followed by a proliferative phase (day 4–14) with angiogenesis and production of extracellular matrix (ECM), mediated by fibroblasts, macrophages and lymphocytes, and a remodeling phase (>day 14) where collagen remodeling increases the strength of the anastomosis. Any perturbation of these processes, including preexisting infection, ischemia, or compromised immunity, can result in AL [11].

To promote anastomotic wound healing, several strategies have been proposed, including (i) the application of external reinforcements, such as fibrin sealants and collagen patches [12], (ii) inhibition of specific matrix metalloproteases [13], (iii) the addition of growth factors [14], and (iv) the usage of cell therapies [15], which have shown promising results, albeit with varying degrees of success. Thus, further studies on understanding the wound healing process of the different segments of the intestine, and the development of treatments that can improve anastomotic healing are necessary.

Silkworm fibroin, which represents the structural-protein component in silk, is an FDA-approved biomaterial with suitable biomechanical properties, high biocompatibility and controllable biodegradation, which makes it a suitable biomaterial for biomedical purposes, including drug delivery, tissue engineering, and promotion of wound healing [16,17,18,19,20,21]. Depending on the application, fibroin can be used to create porous scaffolds, films, nanoparticles, and hydrogels [22]. Recently, a glycidyl methacrylate-modified fibroin hydrogel significantly promoted the repair of meniscus tears in an in vivo rabbit model stimulating an organized cell proliferation and collagen deposition [23], whereas fibroin scaffolds have been shown to facilitate the recovery from full-thickness skin wounds, by enhancing fibroblast adhesion, angiogenesis and regulating extracellular matrix synthesis [24,25]. However, to date, no study has evaluated the effects of fibroin films on the wound healing of intestinal anastomoses. Using a Wistar rat model of colo-colic anastomosis, we set out to analyze the effects of a single layer of fibroin sheets on anastomotic strength, inflammation, and collagen deposition on postoperative day 3 (POD3), representing the early phase of wound healing.

## 2. Materials and Methods

### 2.1. Silk Fibroin Processing and Films Preparation

Cocoons of Bombyx mori were obtained from worms reared in the sericulture facilities of IMIDA (Murcia, Spain). Cocoons were chopped and boiled in 0.02 M Na_2_CO_3_ (for 30 min) to eliminate the sericin. Then, the raw silk fibroin (SF) was rinsed with distilled water and dried at room temperature for 3 days. Subsequently, SF was dissolved in 9.3 M LiBr (Acros Organics, Thermo Fisher Scientific, Waltham, MA, USA) for 3 h at 60 °C, yielding a 20% *w*/*v* dissolution that was dialyzed against distilled water for 3 days (Snakeskin Dialysis Tubing 3.5 KDa MWCO, Thermo Fisher Scientific) with eight total water changes (at 4 °C) [26]. A 6.6% *w*/*v* SF dissolution was retrieved and diluted to a final concentration of 2% *w*/*v* for film fabrication. SF films were produced by casting 700 µL of the diluted solution onto plastic Petri dishes (3.7 cm in diameter), resulting in films approximately 10 µm thick after drying at room temperature for 48 h. The thickness of the films was determined with an electronic digital micrometer (Mitutoyo Digimatic Micrometer 0–25 mm, resolution of 0.001 mm, Mitutoyo Corporation, Kawasaki, Japan). To enhance the beta-sheet content of fibroin and render the films water-insoluble [27], a water annealing process was conducted. This involved placing the dried films in a vacuum desiccator (650 mBar) containing water for 24 h. Finally, the films were placed between filter papers to prevent the formation of undesired folds, then sealed in heat-sealable bags and sterilized by autoclaving.

### 2.2. Animals

Male Wistar rats (10–12 weeks old) were purchased from the Animal Experimentation Unit of the University of Granada (UGR) and housed in makrolon cages (one animal/cage), maintained in an air-conditioned atmosphere with a 12 h light–dark cycle and provided with free access to tap water and food in the animal facilities of the UGR. The study was conducted in compliance with the ARRIVE guidelines 2.0, the “Guide for the Care and Use of Laboratory Animals” issued by the U.S. National Institutes of Health (NIH), in accordance with national and European regulations (RD1201/2005, 32/2007, 2010/63/EU and RD53/2013), and with the approval of the laboratory animal ethics committee (CEEA-1643-2025).

### 2.3. Study Design and Surgical Procedures

All surgical materials were autoclaved before use. Rats were fasted for 24 h preoperatively and anesthetized using isoflurane (Isoflo^®^, Esteve, Barcelona, Spain). The rats were divided into two groups (control (C) or fibroin treatment (F)). Blocks of four animals were operated each day, with two subjects from each experimental group. We generated all possible treatment sequences within the block (e.g., CFCF, FCCF, CCFF, FFCC, etc.) and one particular sequence was randomly selected for each block. This was repeated until achieving the final sample size of N = 11/group. The researchers analyzing the outcome were blinded to the treatment allocation. All rats received a 2.5 cm midline laparotomy to access the abdominal cavity followed by a 1 cm colon resection. All resections were performed to an intestinal section 5 cm distal to the caecum. End-to-end colo-colic anastomosis was performed using 6-0 propylene sutures (Prolene*, ETHICON ENDO-SURGERY GmbH, Norderstedt, Germany) in a single-layer interrupted fashion. Propylene was used as a material because it is a monofilament, non-absorbable suture. Its advantages include high tensile strength, minimal tissue reactivity, and slipperiness, which allows easy removal from tissues. The end-to-end anastomosis was sutured using magnifying loupe glasses. In the fibroin group, a single layer of fibroin membrane was applied externally around the anastomosis. All anastomoses were performed by the same surgeon. The fascial layer of the abdominal wall and the abdominal skin were closed in a continuous pattern of 3-0 Perma-Hand Silk (Ethicon). During the procedure, the animals were kept on a warming blanket at 38 °C. A saline solution was dropped onto the eyes to avoid drying and subsequent irritation of the cornea. No controlled hemorrhagic shock was created during the procedure. Postoperatively, the animals received analgesic (Buprenorphine, 0.05 mg/kg/day, i.p (after the surgical procedure and once daily for two days) (EUMEDICA Pharmaceuticals GmbH, Lörrach, Germany)) and were fed with standard rat food and drinking water. Moist chow food pellets were weighed and added on the cage floor/bedding (50 g) and in the cage lid (100 g) and were weighed again at the end of the experiment to measure food consumption. Rats were monitored at least twice daily for postoperative distress, including assessment of body weight, food intake, fecal output, grooming behavior, mobility, and wound condition.

### 2.4. Assessments

Three days post-operation, which has been described as the period of highest susceptibility to anastomotic failure in this model [11], rats were euthanized using a high dose of anesthesia containing isofluorane for assessments. We proceeded to sacrifice the rats until no heartbeat was detected for at least 5 min. The previous abdominal incision was reopened. After exposure of the cavity, a checklist was followed to search for signs of peritonitis, abscesses, blockage, dehiscence, or leakage of the anastomosis, signs of intestinal obstruction, and adhesions.

A 10 cm segment of the colon with the anastomosis in the middle was resected. Care was taken not to detach adhesions from the anastomosis, but to dissect the surrounding tissues. The resected specimen was gently irrigated with saline to remove feces and was mounted on a table.

#### 2.4.1. Evaluation of Adhesions

At post-mortem examination, adhesions were graded on a scale between 0 and 3 according to the model developed by Evans [28], which distinguishes between “adhesions separated by dissection” (score = 3), “adhesions separated by traction” (score = 2), “self-departing adhesions (score = 1), and “no adhesions” (score = 0).

#### 2.4.2. Measurement of the Colonic Bursting Pressure

The anastomotic bursting pressure was measured in situ, under constant temperature and pressure conditions, by applying internal hydraulic pressure using a mercurial sphygmomanometer, calibrated with a reference standard manometer, as indicated by the manufacturer (Tanaka Sangyo Co., Ltd., Tokyo, Japan). After the integrity of the anastomosis was ascertained upon exposure of the line of anastomosis and removal of adhesions in the surrounding tissues, two cuts, one 5 cm proximal and one 5 cm distal to the anastomosis were made to obtain a 10 cm long colonic segment. The distal end of this segment was closed after a pressure sensor mercury manometer was placed into the proximal end. Subsequently, the colon segment was immersed in saline solution. The pressure was increased gradually and the appearance of air bubbles in saline was recorded as the bursting pressure in mmHg. Bursting pressure was recorded as the peak pressure attained immediately before rupture of anastomosis. Following this measurement, a tissue sample taken from the anastomosis line was sent to be histopathologically evaluated by a single expert pathologist using a conventional light microscope.

### 2.5. Histological Staining and Evaluations

The colo-colic anastomoses were fixed for 24 h in 4% paraformaldehyde and subsequently dehydrated using increasing concentrations of ethanol (70%, 85%, 95% and 100%). The sutures were then removed, and the tissue samples were embedded in paraffin and cut into 5 µm-thick longitudinal sections. For the eosin/hematoxylin/alcian blue staining, the tissue sections were deparaffinized, rehydrated, and incubated with 1% alcian blue in 3% acetic acid for 30 min, followed by undiluted Harris hematoxylin for 4 min (VWR, VWR lnternational Eurolab, S.L.U., Llinars del Valles, Spain), and 0.5% eosin (Merck, Merck Life Science S.L.U., Madrid, Spain) for 45 s. For the Masson’s trichrome stain, the slides were deparaffinized, rehydrated, and treated with Bouin’s fluid at room temperature overnight. The rest of the staining procedure was carried out according to the manufacturer’s instructions (Abcam, Cambridge, UK). Stained slides were then mounted with DPX media (Casa Alvarez, Madrid, Spain).

All tissue slides were scanned on a Pannoramic midi scanner (3DHISTECH Kft, Budapest, Hungary). For the hematoxylin/eosin/alcian blue stainings, the stained slides were evaluated microscopically in a blinded fashion for granulocyte infiltration, monocyte/macrophage infiltration, and the presence of fibroblasts according to the modified Ehrlich–Hunt score, where the abundance of each cell population was graded as 0 = no evidence, 1 = occasional evidence, 2 = light scattering, 3 = abundant evidence and 4 = confluent cells or fibers [29].

The Masson’s trichrome-stained images were analyzed with QuPath [30]. In brief, the anastomoses were defined, and the selected areas were segmented into SLIC superpixels (gaussian sigma = 5; superpixel spacing = 25; numbers of iterations: 25) and annotated according to the stain for collagen (blue), and muscle fibers and cytoplasm (red). The random trees (RTrees) pixel classifier was used to analyze the images (high resolution), and the amount of collagen was represented as % collagen stain of total area.

A schematic illustration summarizing the experimental design and the procedures is shown in Supplementary Figure S1.

### 2.6. Statistical Analysis of Data

The statistical analysis was performed using the GraphPad Prism version 7 software (GraphPad Software, Inc., La Jolla, CA, USA). Data normality was analyzed using the Shapiro–Wilk and the Kolmogorov–Smirnov tests. The comparisons of bursting pressure, tissue adhesion and the Ehrlich–Hunt scores between control and fibroin-treated groups were done using a two-tailed Mann–Whitney U test. The food consumption data was analyzed using the Student’s *t*-test. Data were plotted as mean (standard deviation (SD)). A *p*-value < 0.05 was considered significant.

## 3. Results

### 3.1. Fibroin Sheets Improve the Strength of Colo-Colic Anastomoses on POD3 in Wistar Rats

**Figure 1:**
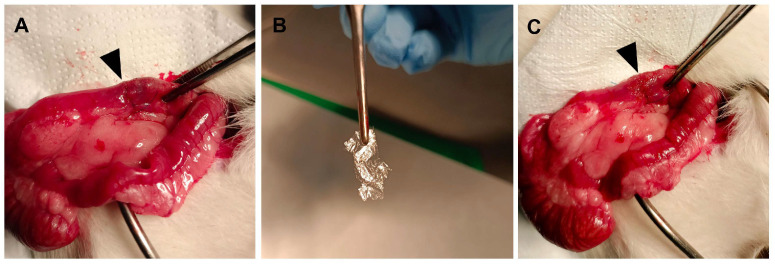
Application of silk fibroin sheets in a rat model of colo-colic anastomosis. (**A**) End-to-end colo-colic anastomosis in a single-layer interrupted fashion was performed as described in the materials and methods. (**B**) Dry autoclaved fibroin patches (10 μm) were used to cover the anastomosis in a single layer. (**C**) Arrow indicating the anastomosis covered with a sheet of silk fibroin.

Rats were subjected to a colo-colic anastomosis (Figure 1A), and a single layer of dry autoclaved 10 μm-tick fibroin sheet was used to cover the anastomosis, allowing it to firmly adhere to the colon (Figure 1B,C). The rats subjected to the anastomotic procedure recovered well in both groups, and no rat died on the day of operation or during the following three days.

**Figure 2:**
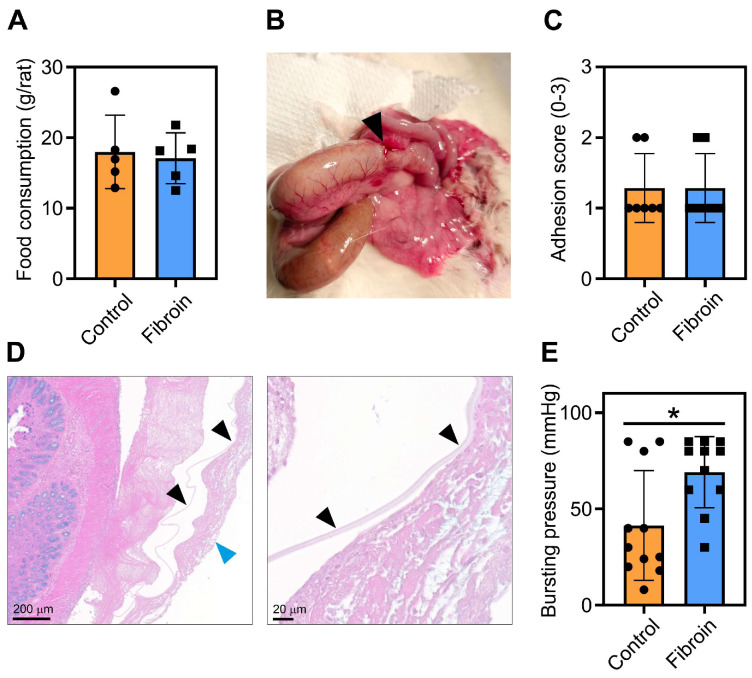
Analysis of food consumption, intestinal adhesions and anastomosis strength, on day 3 post-operation (POD3). (**A**) Food consumption was measured individually for each rat by weighing the pellets before the operation and on POD3 (N = 5/group). (**B**) Example of the gross morphology of an anastomosis (indicated with a black arrowhead). (**C**) Evaluation of tissue adhesions according to Evans et al. [28] (N = 7/group). (**D**) Examples of fibroin sheets (indicated with black arrow heads) in a tissue section stained with hematoxylin and eosin at different magnifications. The blue arrow indicates the external cell layer covering the fibroin membrane. (**E**) The bursting pressure, which gives an indication of the strength of the anastomosis, was measured with a mercury manometer (N = 11/group). Data are shown as mean (SD), with closed circles and boxes indicating individual animals. * = *p* < 0.05.

There was no difference in food intake (Figure 2A), or weight loss between the two groups, and feces were found in all cages. On postoperative day 3 (POD3), a macroscopic evaluation of the anastomoses showed no apparent complications, with good vascularization and no evidence of collections, free fluid, obstruction, or peritonitis, and the fibroin sheet was not apparently visible at the anastomotic site (Figure 2B). Using the Evans score of intra-abdominal adhesions [28], we did not detect any differences between the control or fibroin-treated rats (Figure 2C). Interestingly, the fibroin sheets were apparent in eight out of nine hematoxylin and eosin-stained tissue sections from fibroin-treated rats. Although deformed due to the sectioning, the fibroin material was localized close to the serosa and was in some cases covered with a tissue layer of variable thickness, consisting mainly of fibroblastic cells and the scattered presence of granulocytes (Figure 2D and Figure S2). Finally, measuring the bursting strength of the anastomoses on POD3 showed that the fibroin-covered anastomoses (69 (18) mmHg) were significantly stronger compared to the anastomoses in the control group (41 (28) mmHg) (Figure 2E).

Bursting pressure of the colo-colic anastomoses and adhesion scores were also evaluated on day 6 (POD6), in a small number of rats (N = 4/group). None of the anastomoses treated with fibroin sheets exhibited leakage upon pressure testing, whereas one control anastomosis leaked at 90 mmHg and another animal died due to anastomotic leakage on POD3. No differences in adhesion scores were observed between groups (Supplementary Table S1). However, due to the small sample size and limited analysis of the anastomoses on POD6, no conclusions can be drawn from these data.

### 3.2. Fibroin Does Not Affect the Magnitude of Acute Inflammation Along the Anastomotic Line in Comparison to Controls on POD3

**Figure 3:**
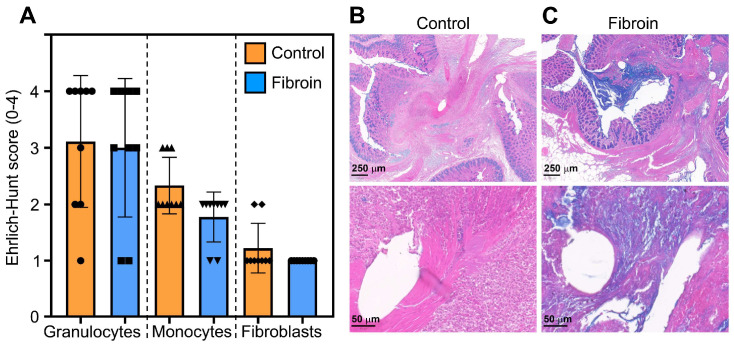
Histological evaluation of the anastomoses from control and fibroin-treated rats on POD3. Formalin-fixed, paraffin-embedded tissue sections were stained with eosin, hematoxylin, and alcian blue. (**A**) The presence of granulocytes, monocytes/macrophages and fibroblasts was graded according to the modified Ehrlich–Hunt score. The data are shown as mean (SD), and the symbols represent individual rats (N = 9 rats/group). Representative images of a control (**B**) and a fibroin-treated (**C**) anastomosis are shown at 1.5× magnification (**upper** panel), and at 11× magnification (**lower** panel), using the QuPath software (version 0.4.3).

Next we analyzed the anastomoses by histochemistry, evaluating the number of granulocytes, monocytes/macrophages and fibroblasts in the wound bed using the eosin and hematoxylin stains, and the amount of collagen fibers using the Masson’s trichrome stain. We found high granulocytic infiltration along the anastomotic line in both control and fibroin-treated rats, which is in line with the acute inflammatory response prevalent on POD3 (Figure 3A–C). Monocyte infiltration and the presence of macrophages in the anastomoses were less pronounced, with levels lower in the fibroin-treated group, although not reaching statistical significance. Finally, we detected low numbers of fibroblastic cells in both groups.

**Figure 4:**
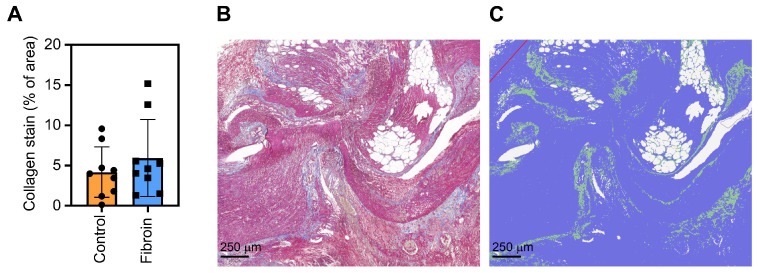
Analysis of the collagen amount in the anastomotic wound beds of control and fibroin-treated rats at day 3 post-operation. (**A**) FFPE tissue was stained with Masson’s trichrome reagent, scanned, and the staining of collagen (blue) was evaluated using the QuPath software. The data are shown as mean (SD), with closed circles and boxes indicating individual animals (N = 9 rats/group). (**B**) Representative image showing the Masson’s trichrome staining of a control anastomosis, showing the collagen stain in blue. (**C**) Example of an annotated image using QuPath with the collagen stain labeled in green, and the rest of the tissue in blue.

Using Masson’s trichrome stain, we evaluated the amount of collagen in the colo-colic anastomoses of control and fibroin-treated rats of POD3. Although there was a trend towards higher collagen content in fibroin-treated anastomoses, no significant differences in collagen amount were observed between the two groups (Figure 4A–C). These data show that the fibroin films do not reduce the acute inflammation in the anastomoses early after the intervention. On the contrary, fibroin-treated anastomoses showed slightly less monocytes and fibroblasts, indicating a slower progression from the acute inflammatory phase to the proliferative phase of the wound healing process (Figure 3).

## 4. Discussion

AL is one of the major complications after colorectal surgery, with a prevalence of 2–20% and an estimated mortality of 4–16% [1,2,31,32]. AL is most prevalent during the first 1–2 weeks after the surgery and is associated with risk factors related to the frailty of patients and tissues, suggesting a perturbed or delayed wound healing of the anastomosis [9]. It is important to identify risk factors and improve surgical techniques, but also to develop and apply treatments that stabilize the anastomosis and promote wound healing during the early wound healing phase following surgery to minimize the risk of AL.

Silk fibroin can be used to create thin films with excellent elasticity and mechanical strength, and both fibroin films and scaffolds have been used successfully to promote wound healing of the buccal mucosa [33], and full-thickness skin wounds in rats [34]. However, no study has evaluated the effect of fibroin sheets on the strength of colon anastomoses in rats. Thus, we proposed that the use of fibroin sheets in colon anastomoses would improve the stability and/or wound healing of the anastomoses early after the intervention, decreasing the probability of suture dehiscence.

In rat models of colon anastomosis, the effects of treatments aiming to increase anastomotic strength are mainly assessed on POD3-7. On POD3, the anastomosis is generally weak and characterized by granulocytic inflammation. However, the progression of wound healing with a gradual increase in fibroblast activation, extracellular matrix maturation, and a reduction in inflammation is seen at POD7 and onwards [11,35]. We chose POD3 as an endpoint to evaluate the effects of the fibroin sheets on anastomotic bursting strength, either due to its role in external stabilization of the anastomosis, or its modulation of the wound healing process, or both. We found that application of a single layer of silk fibroin films increased the stability/healing of the anastomoses, as evidenced by an increased bursting pressure on POD3, in comparison to controls. However, we did not observe any effect on the number of granulocytes and monocytes/macrophages between the two groups on POD3.

Inflammation is a physiological response to injury, and a key process in anastomotic wound healing [11]. Slieker et al. [36] detected a significantly increased incidence of AL in patients on long-term corticosteroid treatment. Similarly, the usage of non-steroidal anti-inflammatory drugs (NSAIDs) during the first days after intervention has been associated with increased AL, although the results are variable [37]. However, many studies have identified pre- and postoperative inflammatory molecules, including C-reactive protein, as risk factors for AL, suggesting that a failure to control the magnitude and duration of acute inflammation can also result in failed wound healing of the anastomosis [38,39]. Although some fibroin-based compositions have anti-inflammatory properties [40], most studies employing silk fibroin products for wound healing purposes have observed a low-grade transient inflammation in response to fibroin, with myeloid cells and multinuclear giant cells found in association with the fibroin [41]. Our data suggest that the fibroin films do not affect the early acute inflammation generated at the anastomosis, which might be beneficial for the wound healing process, since a slow transition from the inflammatory to the proliferative phase supports a more organized extracellular matrix formation and improves subsequent tissue strength.

Next we analyzed the collagen content in the anastomostic wound areas on POD3. Oxlund et al. [42] showed that collagen deposition, as measured by incorporation of tritiated hydroxyproline, in rat colon anastomoses was rapidly increased following surgery, increasing 20-fold on POD4, and reaching a maximum on POD6. In addition, Martens et al. [43] demonstrated that the collagen production, as measured by the incorporation of radiolabeled hydroxyproline, reached its maximum on POD3-4 in rat anastomoses. However, using the Masson’s trichrome staining, we did not observe an increase in collagen content at the anastomotic wounding site between the control and fibroin sheet-group on POD3. This agrees with our histological data, showing similar levels of monocytes and fibroblasts close to the transection line in both groups.

As summarized above, it does not appear that the fibroin sheets accelerate the wound healing on POD3, probably because it is an early stage of the healing process. This suggests that the observed increase in bursting pressure of the fibroin-treated anastomoses might be attributed to its role as an external stabilizer of the anastomosis. Based on our findings and the previous literature, we propose that silk fibroin acts, during the early inflammatory phase, primarily as structural support, providing mechanical reinforcement of the anastomosis, boosting its tensile properties and flexibility while allowing the inflammation to proceed, a process (if not dysregulated by infection or exaggerated tissue damage) which is important for subsequent wound healing. This is in agreement with our observations of fibroin sheets close to the serosa at the anastomotic site, sometimes together with an external cell layer. Although fibroin degrades over time in vivo, studies have shown that fibroin scaffolds have a half-life in rats of 5–10 days in vivo, depending on tissue location [44]. Beyond its mechanical role, silk fibroin exhibits intrinsic bioactivity. Several studies have demonstrated that fibroin and sericin stimulate cell migration and re-epithelialization through activation of MEK, JNK, and PI3K pathways, leading to upregulation and phosphorylation of c-Jun, a key transcription factor in wound healing [45]. More detailed studies on the effect of fibroin on the anastomotic wound healing process, including at later time points (POD7 and later), are necessary.

One possible drawback of using external biofilms/sheets is the potential of bacterial growth underneath, adhering to the biomaterials and causing treatment-resistant infections [46]. We have used autoclaved, sericin-free silk fibroin films extracted using lithium bromide, which increases their biocompatibility and antimicrobial activity, respectively [47], but bacterial contamination/infection still poses a serious risk. Interestingly, several silk fibroin-based biomaterials, including silk microfibrous mats, with antibacterial properties have been generated through their physical loading or chemical functionalization with different antibacterial agents [17,48]. Similarly to SF nanoparticles, in addition to antimicrobial functionalization, it would also be possible to add anti-inflammatory and antioxidant drugs to these fibroin sheets, which could further improve their therapeutic effect on anastomosis healing [49].

Among the limitations of the study are the small sample size and the lack of later time points of analysis of anastomotic wound healing (e.g., day five, seven, and later). Furthermore, we have used a low-risk model of colo-colic anastomosis that does not result in a high incidence of anastomotic leakage. Future studies should evaluate the wound healing properties of silk fibroin films using TNBS-induced colitis or ischemic high-risk colon anastomosis models [50]. However, the silk fibroin films possess several advantages, including easy application, and the material was well tolerated by the rats within the short timeframe of the experiment. This is due to its well-known biocompatibility and its excellent mechanical properties. In addition to this, fibroin can be processed in different configurations and sizes that meet the requirements of the tissue to be repaired or replaced (dissolutions, hydrogels, films, sponges, etc.), and these biomaterials are sterilizable by autoclave, among other techniques [26]. These properties make fibroin a promising choice as an external reinforcement of anastomoses, since other commonly employed wound dressings, such as collagen and elastin, are more difficult to manufacture and possess inferior mechanical properties [51]. In comparison to sealants and glues, the fibroin sheets do not impede the interaction between the two intestinal segments in the anastomosis, which could hinder proper wound healing between the two tissue pieces [52]. Furthermore, the healing-promoting nature of silk fibroin is widely described in the scientific literature, and this characteristic gives it a key role as a biomaterial to be used in this field [45,51,52,53,54].

## 5. Conclusions

In summary, we have shown that a single layer of silk fibroin film applied to colo-colic anastomoses increases their bursting pressure on POD3, without affecting the granulocytic inflammation or accelerating the wound healing process. Further studies on the effect of silk fibroin films on the initial stabilization and the wound healing of colo-colic anastomoses are necessary, but we propose silk fibroin films as another promising biomaterial that could, in the future, be used to decrease the incidence of early anastomotic leakage in patients undergoing colonic surgery.

## Data Availability

The original contributions presented in this study are included in the article/Supplementary Material. Further inquiries can be directed to the corresponding author.

## Supplementary Materials

The following supporting information can be downloaded at: https://www.mdpi.com/article/10.3390/jfb17030126/s1, Supplementary Figure S1: Schematic illustration of the study workflow. Supplementary Figure S2: Representative images of fibroin sheets in tissue sections. Supplementary Table S1: Analysis of control (N = 4) and fibroin-treated (N = 4) rats on day 6 after surgery.
