# Supplementary material for: Silk Fibroin Sheets Improve the Strength of Colon Anastomoses in Wistar Rats
Source: J Funct Biomater. 2026 Mar 4;17(3):126. doi: 10.3390/jfb17030126 (PMC13027351; doi:10.3390/jfb17030126)
Supplement: Supplementary file 1 [file jfb-17-00126-s001.zip › Supplementary Figure Legends.pdf]

Supplementary Figure S1. Schematic illustration of the study workflow showing treatment groups, experimental procedures, and outcome assessments.

Supplementary Figure S2. Representative images of fibroin sheets in tissue sections stained with haematoxylin and eosin at different magnifications.
