# Supplementary material for: Silk Fibroin Sheets Improve the Strength of Colon Anastomoses in Wistar Rats
Source: J Funct Biomater. 2026 Mar 4;17(3):126. doi: 10.3390/jfb17030126 (PMC13027351; doi:10.3390/jfb17030126)
Supplement: Supplementary file 1 [file jfb-17-00126-s001.zip › Supplementary Table S1.pdf]

Supplementary Table S1. Analysis of control (N=4), and fibroin-treated (N=4) rats on day 6 after surgery. Bursting pressure of the colocolic anastomoses and the adhesion scores were evaluated as described in the materials and methods. [a)] The pressure limit was reached for the manometer (200 mmHg) with no signs of leakage. [*)] The control rat suffered an anastomotic leakage and died on day 3 post surgery.

Rat ID	Bursting pressure	Adhesion score
Control 1	No leak[a]	2
Control 2	No leak[a]	2
Control 3	-*	-
Control 4	90 mmHg	2
Fibroin 1	No leak[a]	2
Fibroin 2	No leak[a]	2
Fibroin 3	No leak[a]	2
Fibroin 4	No leak[a]	2
