# Supplementary figures and images for: Silk Fibroin Sheets Improve the Strength of Colon Anastomoses in Wistar Rats
Source: J Funct Biomater. 2026 Mar 4;17(3):126. doi: 10.3390/jfb17030126 (PMC13027351; doi:10.3390/jfb17030126)

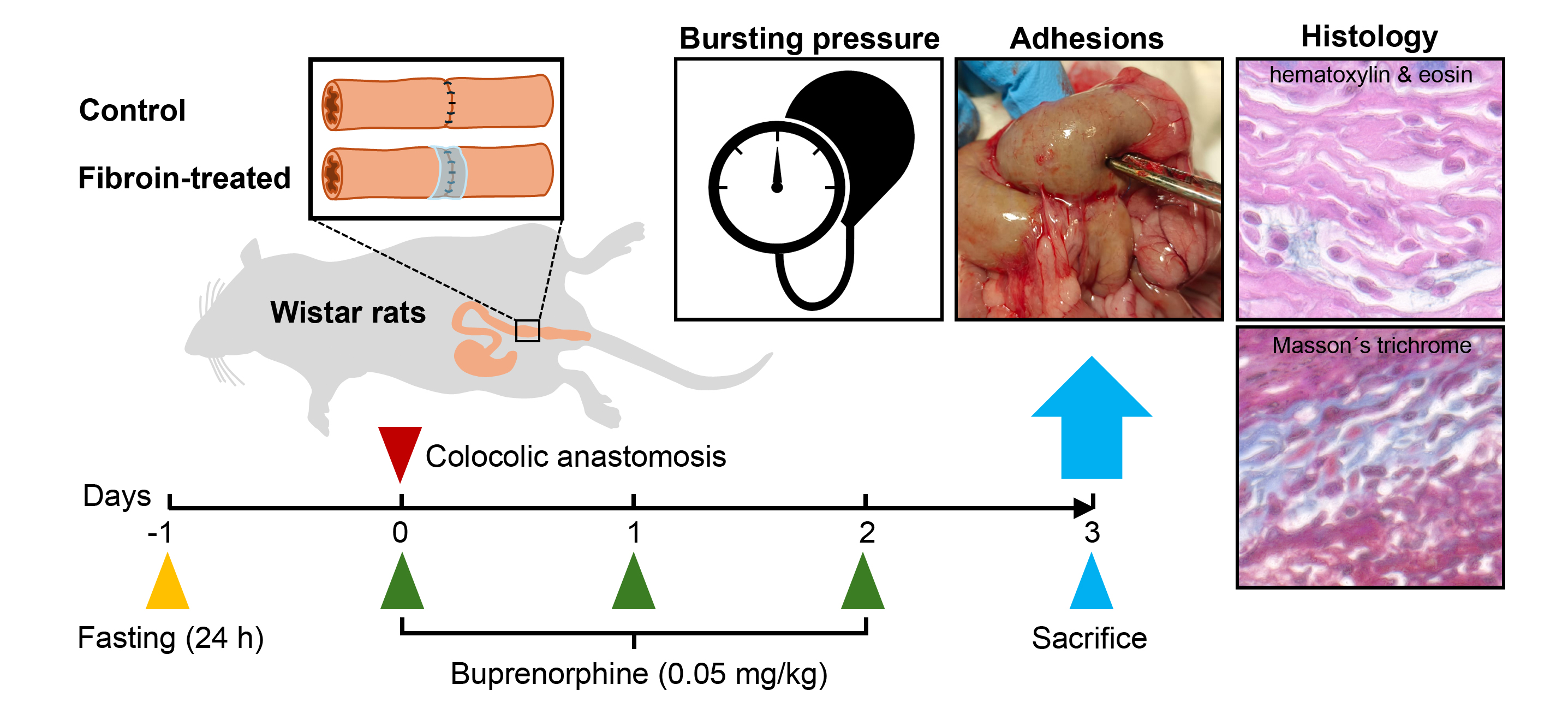

Supplement: Supplementary file 1 [file jfb-17-00126-s001.zip › Supplementary Figure S1.jpg]

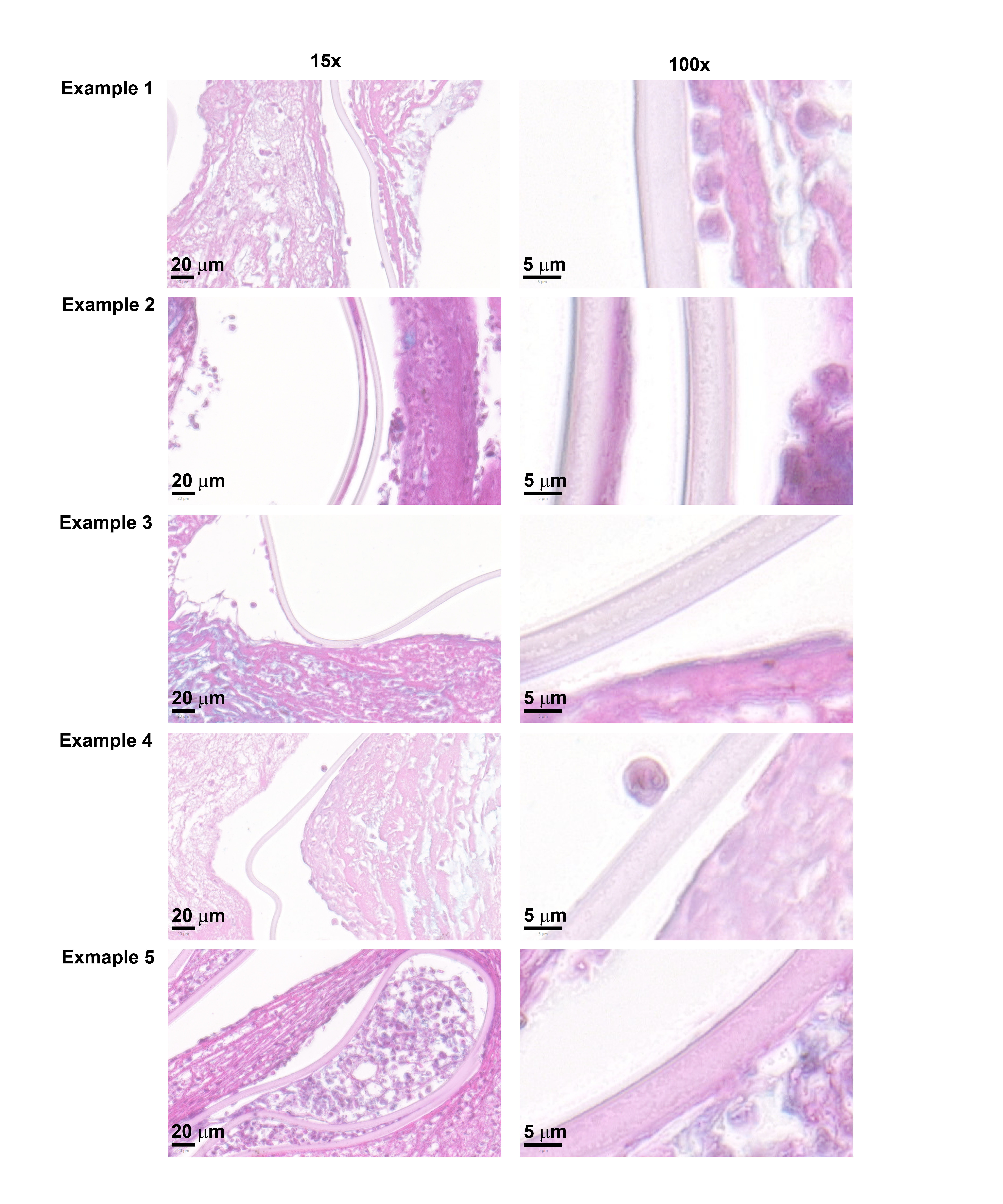

Supplement: Supplementary file 1 [file jfb-17-00126-s001.zip › Supplementary Figure S2.jpg]
